# Generation of peptide detectability datasets from single DIA experiment for prediction model fine-tuning

**DOI:** 10.1093/bioadv/vbag180

**Published:** 2026-06-25

**Authors:** Léo Schneider, Julie Flecheux, Zied Bouyahia, Stéphane Derrode, Jérome Lemoine

**Affiliations:** Université Claude Bernard Lyon1, ISA, UMR5280, CNRS, ISA, Villerbanne, Rhone-Alpes 69100, France; Ecole Centrale de Lyon, CNRS, INSA Lyon, Universite Claude Bernard Lyon 1, Université Lumière Lyon 2, UMR5205, LIRIS, Ecully, Rhone-Alpes 69130, France; Université Claude Bernard Lyon1, ISA, UMR5280, CNRS, ISA, Villerbanne, Rhone-Alpes 69100, France; Ecole Centrale de Lyon, CNRS, INSA Lyon, Universite Claude Bernard Lyon 1, Université Lumière Lyon 2, UMR5205, LIRIS, Ecully, Rhone-Alpes 69130, France; Ecole Centrale de Lyon, CNRS, INSA Lyon, Universite Claude Bernard Lyon 1, Université Lumière Lyon 2, UMR5205, LIRIS, Ecully, Rhone-Alpes 69130, France; Université Claude Bernard Lyon1, ISA, UMR5280, CNRS, ISA, Villerbanne, Rhone-Alpes 69100, France

## Abstract

**Motivation:**

Accurate prediction of peptide detectability in mass spectrometry-based proteomics is critical for improving both protein identification and quantification. Current models generally estimate detectability from amino acid sequences; however, peptide detectability is influenced by the instruments, acquisition methods, and experimental conditions, limiting the applicability of sequence-based models. State-of-the-art approaches mitigate this issue by fine-tuning models for each experimental setup, yet this strategy demands extensive training datasets—often comprising up to 300 000 peptides—incurring substantial experimental and computational costs.

**Results:**

In this study, we present a complementary approach for generating peptide detectability datasets directly from a single DIA experiment. These datasets enable fine-tuning of prediction models with minimal raw data, while improving adaptation to specific experimental conditions. This strategy substantially reduces both the data and cost requirements typically associated with model training. Furthermore, we show that filtering search libraries based on predicted detectability increases peptide identification rates and decreases computational time.

**Availability and implementation:**

Code is available on https://github.com/leoschn/Detectability. Data are available via ProteomeXchange with identifier https://proteomecentral.proteomexchange.org/cgi/GetDataset?ID=PXD076276PXD076276.

## 1 Introduction

Liquid chromatography coupled with high-resolution mass spectrometry (LC-MS) is the cornerstone of bottom-up proteomic analysis, enabling broad and quantitative identification of proteins within complex proteomes. Until recently, the stochasticity of the precursor ion selection and fragmentation step in data-dependent acquisition (DDA) mode hampered the reproducibility of peptide sampling, particularly for peptides derived from low-abundance proteins. Thanks to recent remarkable advances in high-frequency acquisition and data processing (Demichev *et al*. 2020), data-independent acquisition (DIA) mode has revolutionized proteomic analysis by delivering a greater number of protein identifications while reducing analysis times and presenting better coefficients of variation than those obtained in DDA mode.

These significant improvements result from the unbiased sampling mode of DIA, which relies on the successive transmission of isolation windows covering the domain of interest of precursor ion *m*/*z* ratios, usually in the 500–1200 *m*/*z* range.

Although DIA minimizes variability arising from inconsistent spectral scanning and fragmentation, some variability remains due to uneven peptide detection and fluctuations in signal intensity (Nordström *et al*. 2008, Jarnuczak *et al*. 2016). Peptides with a low probability of detection are unreliable as analytical indicators, their presence in a database can negatively affects the detection of detectable peptides.

Whether in DDA or DIA mode, true discovery of peptides for inferring protein identification and quantification are statistically filtered above a False Discovery Rate (FDR) threshold, usually set at 1%. The FDR is selected according to the Peptide Spectrum Matching (PSM) scores, calculated for putative true discoveries and putative false discoveries distributions based on the target decoy approach (Burger 2018). It is important to understand that the *q*-value score of PSM on which the FDR is based depends upon the dataset in which the PSM occurs (Käll *et al*. 2008). In DIA mode, large proteome DIA libraries cause a large query space that likely favors the occurrence of false positives and compromises detection sensitivity (Rosenberger *et al*. 2017, Siyal *et al*. 2021). It is therefore beneficial to optimize the size of the library by eliminating putative peptides that are highly unlikely to be detected due to their intrinsic physicochemical characteristics, known as “non-flyers,” while retaining only the “best flyers.” Since the early 2010s, various factors affecting detectability such as digestibility, ionization potential, and acquisition scheme have been investigated, initially using traditional machine learning approaches (Xu *et al*. 2010). More recently, significant advances have been achieved through the application of deep-learning and sequence-based models (Yang *et al*. 2023a, Abdul-Khalek *et al*. 2025).

In this paper, we propose a method to reduce the amount of raw data required for fine-tuning deep learning models for peptide detectability prediction. Our approach enables adaptation of a model to a new LC-MS setup using just a single DIA experiment to construct the fine-tuning dataset. The paper is structured as follows: after covering the related literature, we describe our method for measuring peptide detectability; next, we demonstrate how it can be used to generate a fine-tuning dataset for a detectability prediction model from a single DIA experiment; finally, as an application, we show how the fine-tuned model can be used to optimize the search library in a proteomics analysis pipeline.

## 2 Related work

Over the past two decades, peptide detectability—defined as the probability that a given peptide will be successfully identified in an LC-MS experiment—has emerged as a critical determinant of the performance of proteomics workflows (Mallick *et al*. 2007). The challenge arises from the complex nature of the LC-MS pipeline, where numerous factors, spanning from sample preparation to data analysis, contribute to variability in peptide identification rates.

The importance of accurate peptide detectability prediction goes beyond academic interest, directly influencing practical applications such as biomarker discovery, the development of targeted proteomics assays, and data-independent acquisition (DIA) workflows. Recent advances in machine learning, particularly deep learning architectures, have significantly improved detectability prediction, achieving unprecedented accuracy while also highlighting challenges related to model generalizability and cross-platform transferability.

Peptide detectability is determined by a complex interplay of biochemical and experimental factors spanning the entire LC-MS workflow. Variability is introduced during sample preparation, including protein extraction efficiency and enzymatic digestion, with missed cleavages representing a particularly significant source of variation depending on the protocol used (Tang *et al*. 2006). Incorporating predicted missed cleavages and digestibility features has been shown to enhance detectability prediction compared with sequence-only models, yielding measurable improvements in Area Under the Curve (AUC) in recent studies (Yang *et al*. 2023a).

Chromatographic separation introduces additional challenges due to peptide co-elution, where multiple peptides elute at similar retention times, causing signal interference and reduced separation efficiency (Mallick *et al*. 2007). This issue is particularly pronounced in complex biological matrices and can severely limit the detection of low-abundance peptides, thereby compromising both sensitivity and accuracy. Recent studies have demonstrated that accurate retention time prediction models, including those designed for modified peptides such as DeepLC can help reduce identification ambiguity and support MS1-only workflows (Bouwmeester *et al*. 2021).

Ionization efficiency is highly dependent on peptide structure, the ionization method, and the presence of co-eluting peptides competing for available protons (Krokhin 2012). During the detection phase, especially in MS/MS analysis, the chosen fragmentation method strongly influences which peptides and fragments are generated and subsequently detectable. Finally, the software used to extract identifications from raw spectra plays a critical role, as different software packages or variations in analysis parameters can lead to substantially different identification outcomes.

All of the factors mentioned above make detectability an indicator specific to a particular instrument or pipeline rather than a universal property of peptides. The diversity and complexity of these factors make it impossible to fully incorporate them into a predictive model.

A major limitation of current peptide detectability prediction approaches lies in the considerable variability introduced by differences in instruments and experimental protocols (Yang *et al*. 2023b). This heterogeneity significantly hampers the transferability of models across laboratories and experimental setups, posing one of the field’s most pressing challenges. Moreover, variations in protein and peptide abundance can influence identification probabilities and introduce biases in models trained on synthetic libraries, unless appropriate corrections or fine-tuning are applied for specific experimental contexts (Odrzywolek *et al*. 2022). Over the past two decades, the computational strategies for peptide detectability prediction have evolved substantially. Early methods relied on statistical scoring approaches and traditional machine learning techniques, such as fully connected neural networks, decision trees, and support vector machines (Webb-Robertson *et al*. 2008). These models depended on hand-crafted features derived from peptide sequences, requiring considerable domain expertise to select and engineer relevant input variables.

The advent of deep-learning architectures tailored to biological sequences has marked a paradigm shift in peptide detectability prediction. Recurrent Neural Networks (RNNs), followed by Gated Recurrent Units (GRUs) and Long Short-Term Memory (LSTM) networks, enabled the direct use of raw amino acid sequences as model inputs, thereby removing the need for complex manual feature engineering (Hochreiter and Schmidhuber 1997). This development allows models to automatically learn relevant sequence patterns and dependencies that were previously challenging to capture with hand-crafted features.

State-of-the-art models for peptide detectability prediction have increasingly incorporated advanced architectural innovations and multi-modal inputs. DeepDetect uses bidirectional LSTM architectures to integrate sequence and digestibility signals, achieving substantial improvements over sequence-only predictors (Yang *et al*. 2023a). DbyDeep extends this approach by encoding cleavage context, demonstrating that the immediate sequence environment around cleavage sites provides predictive information beyond the peptide sequence itself (Son *et al*. 2023). Recent developments have embraced transformer architectures and protein language models to capture complex sequence dependencies. Methods such as PD-BertEDL and DeepPD leverage BERT-derived embeddings and large-scale protein representations, analogous to evolutionary scale modeling style encodings, to extract richer semantic and evolutionary features from peptide sequences (Wang *et al*. 2022, Li *et al*. 2025). Multi-modal learning has emerged as a particularly powerful strategy. MSBooster integrates predicted retention times, ion mobility, and MS/MS spectra into re-scoring workflows, consistently improving peptide identification across diverse proteomics datasets (Yang *et al*. 2023b). Similarly, DeepDIA combines multiple predictive modalities to enhance spectral library generation and search performance (Rosenberger *et al*. 2014), while Pfly further advances model transferability across experimental contexts through learned embeddings (Abdul-Khalek *et al*. 2025).

## 3 Methods

In the state-of-the-art method pFLy (Abdul-Khalek *et al*. 2025), peptide detectability feature is evaluated using two complementary strategies. The first method is related to the measured intensity of a library of synthetic peptides. It is assumed here that peptide chemical synthesis in a 96-well plate format led to an equimolar mixture. Which is probably not accurate, since it is well known that synthesis yield is determined by the amino acid sequence. The ProteomeTools dataset extracted from the PRIDE repository with the identifiers PXD004732, PXD010595, and PXD021013 was generated according to this principle. The second strategy evaluates peptide detectability feature based on the proportion of samples in which peptides are observed among multiple samples. For instance, the Wang dataset(ProteomeXchange: PXD010154) was generated by analyzing 29 healthy human tissues from the Human Protein Atlas Project. In this framework, the detectability of each peptide is calculated as the number of tissues in which it was identified divided by 29. This approach assumes that every peptide is present in all 29 tissues; however, Wang *et al*. (2019) reported that only 39% of genes are expressed at the protein level across all tissues. While detectability contributes to the non-detection of the remaining 61%, it is not the sole factor. Protein expression varies between tissues—e.g. between brain and kidney—so peptides that are intrinsically detectable may be not detected in tissues in which the proteins they derived from are weakly or not expressed. Similarly, the Sinitcyn dataset (Sinitcyn *et al*. 2023) was derived from bottom-up proteomics analyses of six human cell lines (ProteomeXchange: PXD024364). In this dataset, peptide detectability feature is defined as the fraction of cell lines in which a given peptide was identified.

Herein, to address the limitations of previous approaches, we propose an alternative metric for peptide detectability feature, $I_{\text{Det}}$, which mitigates bias arising from variable protein expression across samples. We define the detectability of proteotypic peptides as the MS2 intensity ($I_{{\text{MS}}^{2}}$) normalized by the maximum intensity of all proteotypic peptides from the same protein ($P_{\text{Prot}}$), as shown in Equation (1). MS2 intensity is directly computed from DIA-NN output as the sum of fragment intensity (column *Fragment.Quant.Raw* of the main output). When multiple precursors share the same sequence (different charges), the maximal intensity among them is kept. This approach is based on the assumption that proteotypic peptides originating from the same protein are present in equal amounts, proportional to the protein’s abundance. Under this assumption, differences in their relative intensities primarily reflect digestibility (e.g. missed cleavages) and inherent detectability. Peptides with the highest intensity within a protein are classified as *strong flyers*, whereas those with lower intensities are considered *weak flyers*. Unlike some previous studies (Yang *et al*. 2021), we do not attempt to disentangle the contributions of digestibility and ionization efficiency, as our metric relies solely on observed intensities. Therefore, we refer to this measure exclusively as peptide detectability. Consequently, this measurement should not be considered an intrinsic property of the peptide, but rather depends on the experimental setup, the analysis pipeline and the protein in which the peptide is found (which impacts digestibility due to miscleavage propensity).


(1) $$I_{\text{Det}}(\mathrm{Pep})=\frac{I_{{\text{MS}}^{2}}(\mathrm{Pep})}{\underset{\mathrm{Pep}\in P_{\text{Prot}}}{\max}(I_{{\text{MS}}^{2}}(\mathrm{Pep}))}$$

Our method significantly reduces the amount of raw data required to infer peptide detectability. A single data-independent acquisition (DIA) experiment is sufficient to generate a detectability dataset containing, on average, 40 000 peptides. Furthermore, any generic DIA experiment can be used, enabling the reuse of data initially acquired for other purposes to construct detectability datasets. We applied this approach to data acquired on two different instruments: a ZenoTOF 7600+ (Sciex) and an Orbitrap Astral (Thermo Fisher Scientific). Each dataset was generated from a single DIA experiment and analyzed using DIA-NN (Demichev *et al*. 2020). Once peptide detectability feature is computed, we infer labels in three different manners. The most straightforward one is to directly use the detectability for regression task. Alternatively, to replicate pFly dataset we classify null detectability peptides (undetected peptides) as non-flyers, while ordering flyers by detectability and label the less detectable third as weak flyer, the intermediate third as intermediate flyer and last third as strong flyer. The last method is to reduce the number of classes to two by aggregating all flyer classes. This last method is coarser and does not rely on quantification. Detailed information on sample preparation, data acquisition, and analysis is provided in Supplementary, available as supplementary data at *Bioinformatics Advances* online. These datasets are hereafter referred to as the *Zeno* and *Astral* datasets, respectively. The Zeno dataset includes 5148 flyers and 1714 non-flyers, whereas the Astral dataset comprises 20 031 flyers and 6675 non-flyers. All mass spectrometry proteomics data have been deposited in the ProteomeXchange Consortium via the PRIDE (Perez-Riverol *et al*. 2025) repository under the dataset identifier PXD076276.

## 4 Results

**Figure 1:**
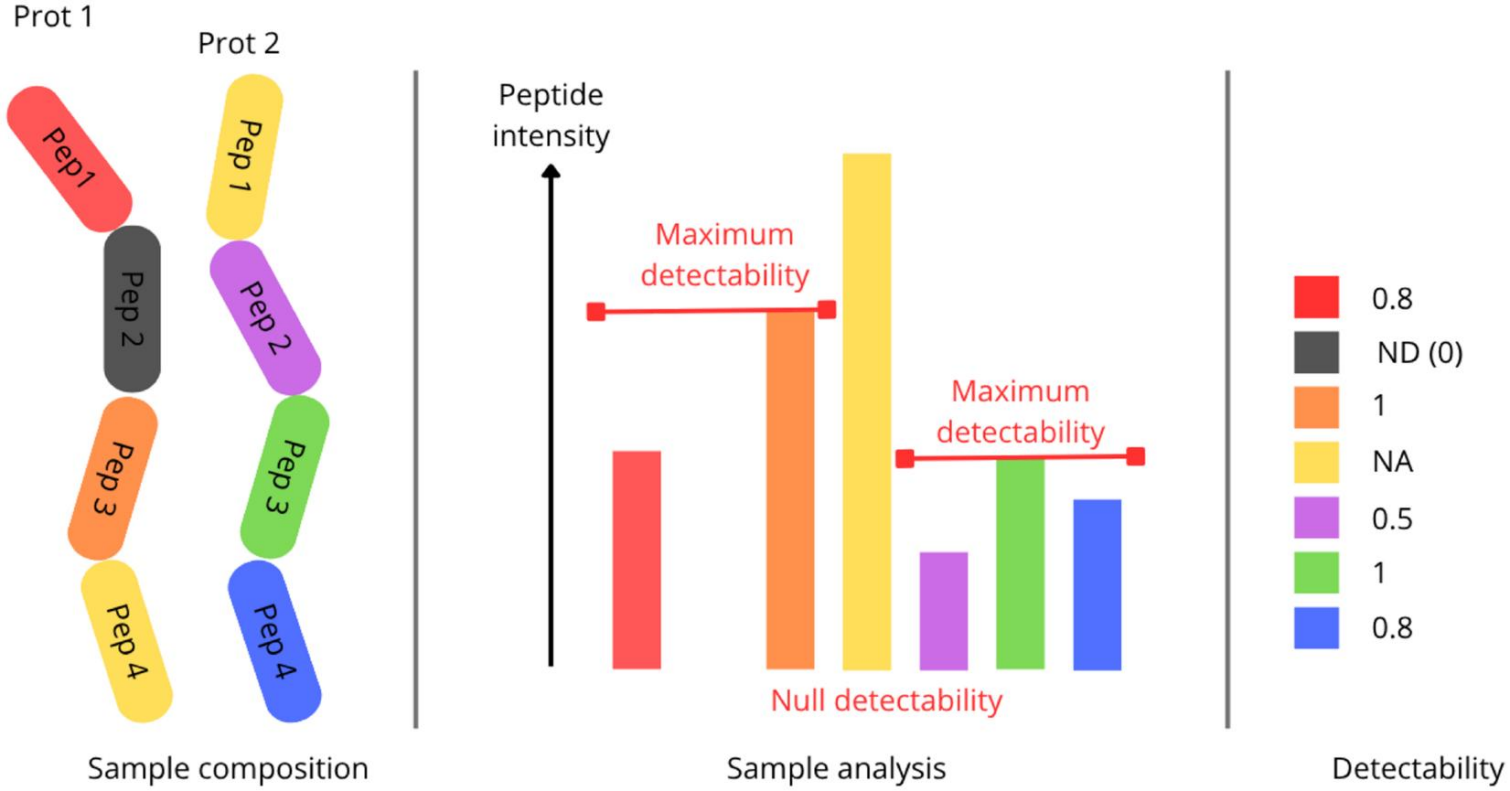
Detectability measurement method. Peptides sharing the same color correspond to identical sequences. Detectability is calculated as the relative intensity of each proteotypic peptide within its parent protein. Non-detected peptides (ND) are assigned an intensity of 0. Non-proteotypic peptides are excluded from the analysis (NA: not applicable).

In this section, we present the results of our experiments. We first examine how peptide detectability varies across different acquisition setups. These variations also affect the performance of prediction models, which do not transfer well between setups. We then show how transferability can be improved by using our detectability dataset generation methods for fine-tuning. Finally, we illustrate the approach with an application case: reducing a search library in a peptide identification pipeline (Fig. 1).

### 4.1 Datasets comparison

**Figure 2:**
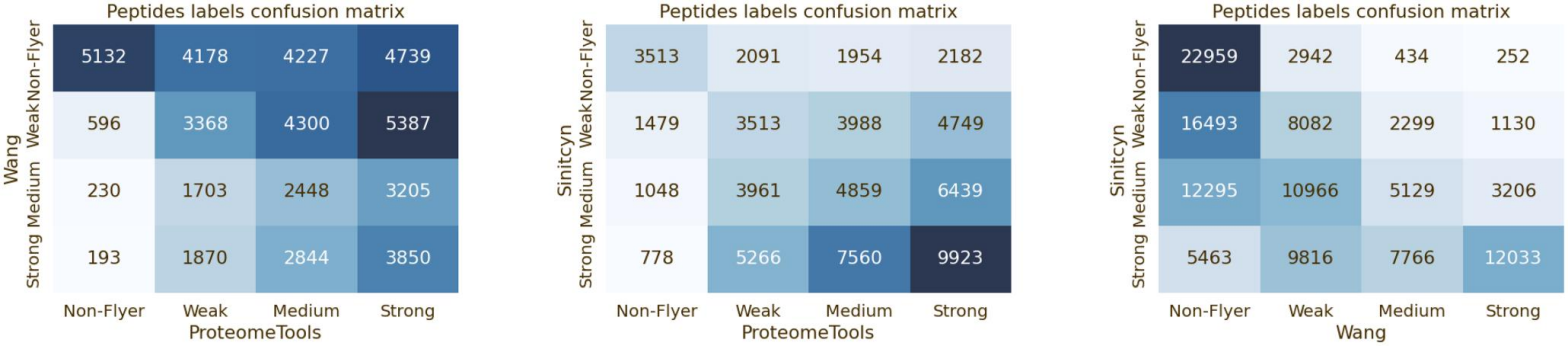
Confusion matrices of peptide labels across different datasets. Left: intersection of ProteomeTools and Wang datasets; middle: intersection of ProteomeTools and Sinitcyn datasets; right: intersection of Wang and Sinitcyn datasets.

Direct comparison of the ground truth labels for peptides shared across multiple datasets highlights how detectability can vary depending on the experimental setup and labeling method. To quantify this variation, we compared the ground truth labels of peptides present in the three datasets introduced in the Pfly study (ProteomeTools, Wang, and Sinitcyn). Confusion matrices summarizing these comparisons are shown in Fig. 2. These matrices are far from diagonal. Only 30.66% (14 798/48 270) of peptides in the intersection of the ProteomeTools and Wang datasets share the same label, increasing to 34.45% (21 808/63 303) for the intersection of ProteomeTools and Sinitcyn, and 39.75% (48 203/121 265) for the intersection of Wang and Sinitcyn (compared with 25% expected for random labels). These results demonstrate that peptide detectability strongly depends on both the acquisition setup and the measurement method. Notably, there is slightly higher agreement between the Wang and Sinitcyn datasets than between either of these and ProteomeTools. This is consistent with the fact that Wang and Sinitcyn are based on biological peptides, whereas ProteomeTools relies on synthetic peptides, suggesting that synthesis-related biases may contribute to these differences. We were unable to perform the same comparison for our datasets due to the limited overlap with the other datasets.

Considering the substantial variability in measured detectability revealed by this comparison, it is unsurprising that no peptide detectability prediction model can achieve universal accuracy across all experimental setups. Consequently, fine-tuning the model with a dataset specific to each setup is crucial for effectively transferring performance between experimental contexts.

### 4.2 Base model evaluation

As a baseline, we evaluated the pre-trained Pfly model on both the Zeno and Astral datasets. Unless stated otherwise, a minimum of four peptides per protein was required for inclusion. This threshold was chosen to reduce normalization bias and eliminate ambiguous identifications. For each dataset, we included all flyers and selected non-flyers (originally in excess) to obtain four roughly balanced classes. The data were split into 80% for training 10% for validation and 10% for testing. This split is done at protein level to avoid information leakage through the normalization used to compute detectability. Due to this constraint class distribution is not strictly equal in across splits. We used the model provided by the authors, which was pre-trained on the ProteomeTools dataset (Zolg *et al*. 2017) and fine-tuned on the Sinitcyn dataset (Sinitcyn *et al*. 2023), acquired on an Orbitrap Fusion Lumos instrument.

To assess the performance of each model, we evaluated recall, specificity, binary accuracy, and categorical accuracy. Recall measures the model’s ability to correctly identify flyers, whereas specificity quantifies its ability to correctly identify non-flyers. The metrics are defined as follows, where *TP* denotes true positives, *TN* true negatives, *FP* false positives, and *FN* false negatives. For all metrics except categorical accuracy, the flyers subclasses (weak, intermediate, and strong) were merged into a single flyer category. The selection of these metrics aligns with those used in pFLy, ensuring a fair and consistent basis for comparison across models.


(2) $$\mathrm{Accuracy}=\frac{TP+TN}{TP+TN+FP+FN}$$

(3) $$\mathrm{Recall}=\frac{TP}{TP+FN}$$

(4) $$\mathrm{Specificity}=\frac{TN}{TN+FP}$$

The base model exhibited poor performance on the evaluated datasets, achieving accuracies of 32% on Astral dataset and 29% on Zeno dataset. For comparison, a random baseline would yield an accuracy of 25%. These findings are consistent with the dataset comparison presented earlier and indicate that the pFly model’s performance does not readily transfer to our LC-MS setup. This underscores the necessity of fine-tuning detectability models to adapt them to LC-MS configurations different from those used during training (Thermo Orbitrap Fusion Lumos), such as the Sciex ZenoTOF and Thermo Astral instruments.

### 4.3 Fine-tuning and transferability

To adapt pFly to our experimental setup, we performed fine-tuning using our datasets. Each model was trained for 50 epochs with the default training parameters recommended by the pFly authors. The fine-tuned models were then evaluated on independent test datasets. For comparison, we also conducted training runs from scratch, with a higher learning rate (5e-2) to ensure model convergence. In addition, we created a combined dataset by merging the Astral and Zeno datasets to assess potential benefits of joint training. Detailed training parameters are provided in Supplementary, available as supplementary data at *Bioinformatics Advances* online.

The results obtained on the Zeno dataset (models trained with various training datasets and tested on the Zeno test set) are presented in Fig. 4. We monitored training and validation curves and did not observe clear signs of overfitting. As previously observed, the base model shows very poor performance. Here, categorical accuracy is the key metric. Fine-tuning the model on the Zeno dataset itself yields the most significant improvement, reaching 43.3% accuracy compared to 31.2% for the base model. Fine-tuning on the Astral dataset provides moderate gains, while training on the combined dataset results in intermediate performance. The training from scratch achieves similar results in comparison to the fine-tuned one (slightly higher categorical accuracy but lower binary metrics) but is more sensitive to hyperparameters especially learning rate. A specific hyperparameter optimization was needed for the model to converge. We provide more details about this optimization in the appendix. While overall performance remains below optimal, it approaches the state-of-the-art results achieved by pFly on the Sinitcyn dataset (47% categorical accuracy).

**Figure 3:**
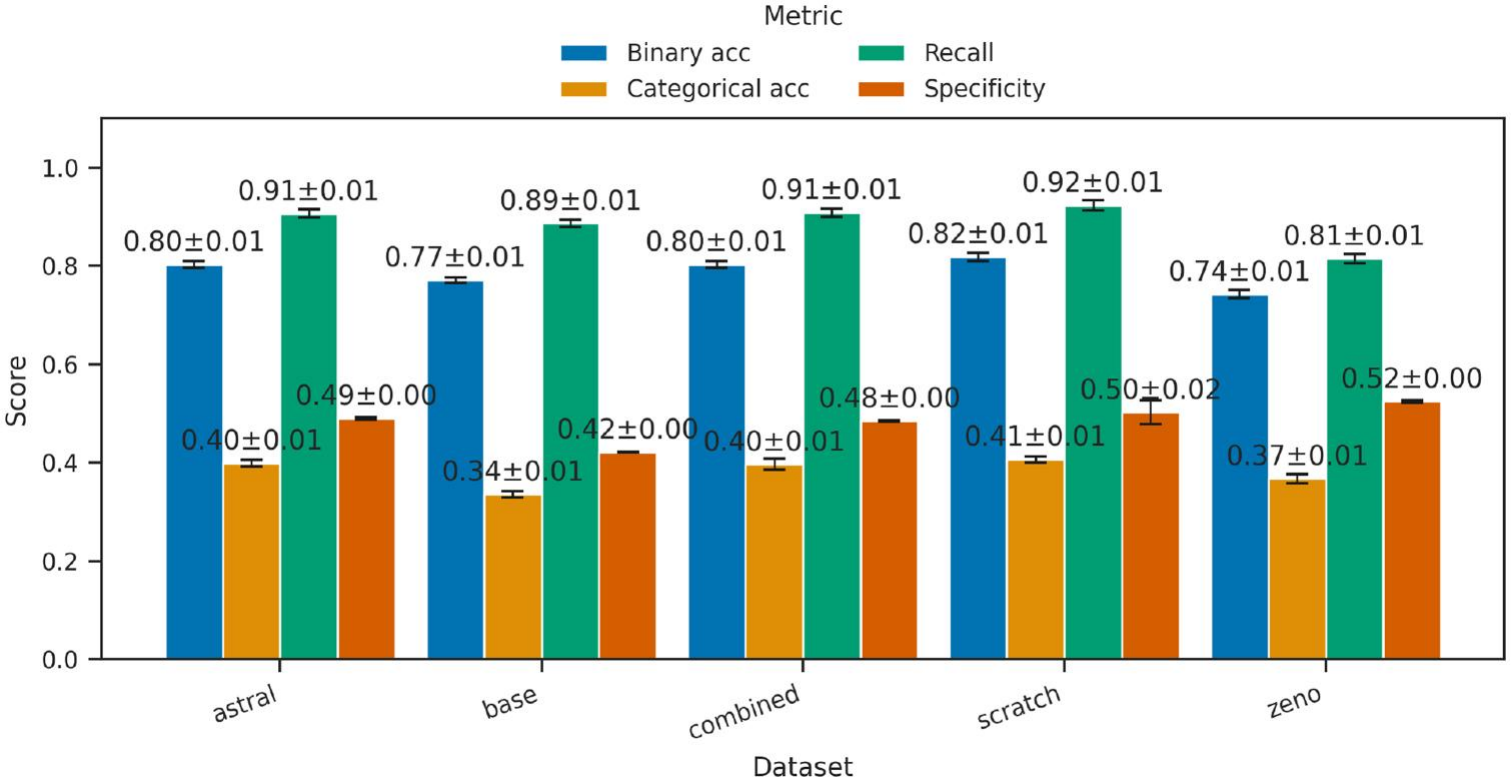
Performance of different pFly-based models on the Astral dataset. Base refers to the original detectability fine-tuned model from the pFly paper; Zeno and Astral correspond to models fine-tuned on their respective datasets. Combined denotes a model fine-tuned on the merged Zeno and Astral datasets, while Scratch refers to a model trained from scratch on the Astral dataset. All models share the same architecture; only the training protocol differs. For each model, binary accuracy (with strong, intermediate, and weak flyer classes merged), categorical accuracy, recall, and specificity are displayed.

On the Astral dataset (models trained with various training datasets and evaluated on the Astral test set), the results shown in Fig. 3 reveal similar trends to those observed for Zeno, with a slight overall decrease in performance. In terms of accuracy, the base model again performs the worst (33.5%), while the best results are obtained by training from scratch on the same dataset used for testing (40.7%). Some noteworthy differences emerge. The performance gap between the model trained on the combined dataset than only on its dedicated one is smaller for Astral dataset than for Zeno dataset (39.7% versus 39.9% for Astral dataset, compared to 40.9% versus 44.4% for Zeno dataset). This may be attributed to the difference in dataset sizes—approximately 23 000 unique peptides for Astral dataset versus 5000 for Zeno dataset. Leading to a combined dataset closer to the Astral dataset than the Zeno dataset.

**Figure 4:**
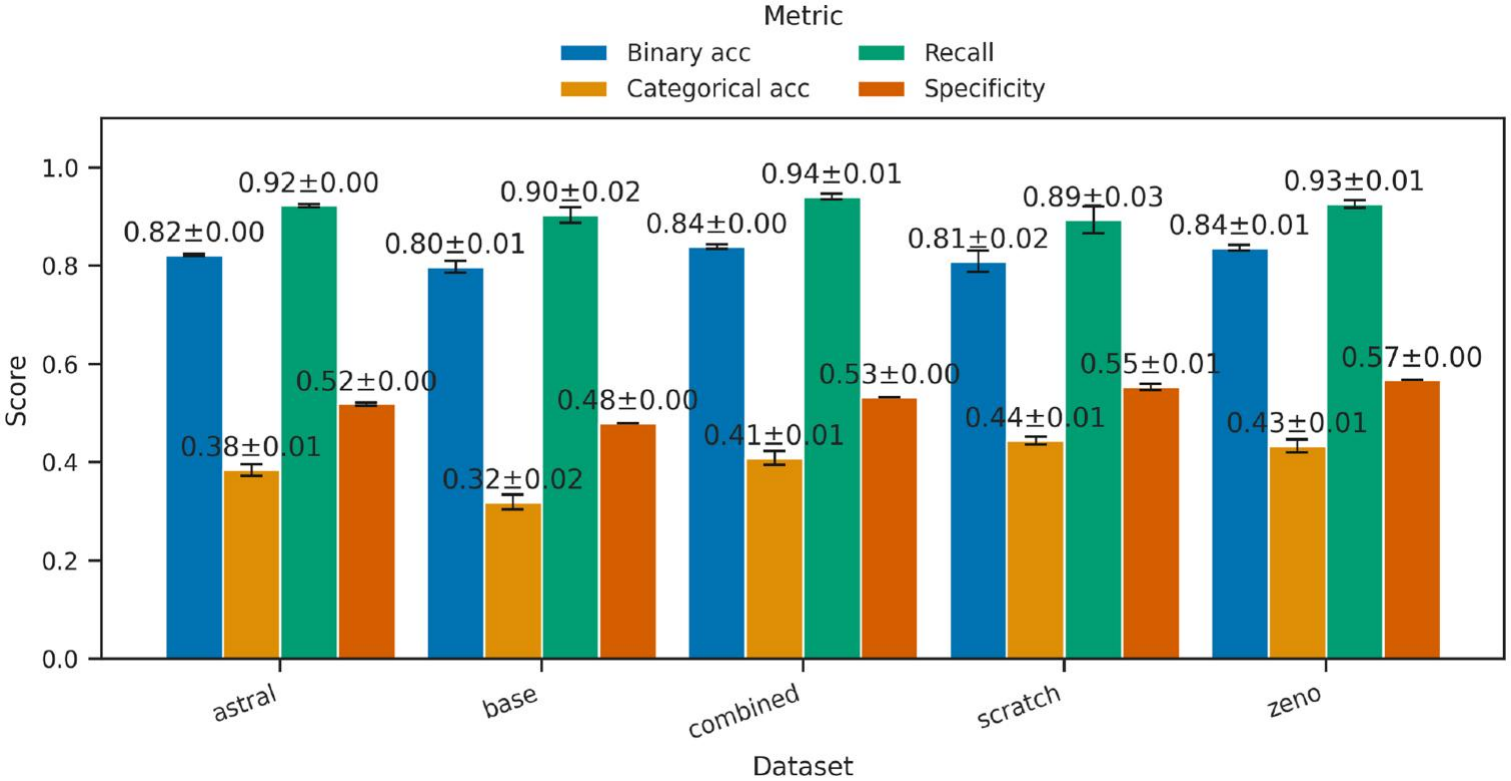
Performance of different pFly-based models on the Zeno dataset. Base refers to the original detectability fine-tuned model from the pFly paper; Zeno and Astral correspond to models fine-tuned on their respective datasets. Combined denotes a model fine-tuned on the merged Zeno and Astral datasets, while Scratch refers to a model trained from scratch on the Zeno dataset. All models share the same architecture; only the training protocol differs. For each model, binary accuracy (with strong, intermediate, and weak flyer classes merged), categorical accuracy, recall, and specificity are displayed.

In summary, both experiments demonstrate that the best-performing configuration is obtained when the pre-trained model is trained from scratch or fine-tuned on the same dataset used for testing. Compared to the base pre-trained model, this setup yields substantial improvements in overall accuracy—particularly categorical accuracy—as well as in specificity. In practice, this means that subclass predictions among flyers are more accurate, and peptides predicted as non-flyers are more reliably identified as such. The fine-tuning approach effectively combines the advantages of a model pre-trained on large and diverse datasets (ProteomeTools and Sinitcyn) with fine-tuning on data acquired using the same instrument (and ionization conditions) as the test set and is less sensitive to hyperparameters than training the model from scratch. This configuration will therefore be adopted as the default for the remainder of this study.

### 4.4 Alternative fine-tuning tasks

Our labeling method uses four distinct classes, which does not perfectly capture the relative intensity measurements. Specifically, we classify flyers into three strictly separated categories, even though the boundaries between adjacent classes are often fuzzier. To investigate the impact of this design choice, we experimented with modifying the labels and, consequently, the fine-tuning tasks. Both alternative approaches remove the arbitrary flyer classes: one merges all flyers into a single class, while the other uses their relative intensity values directly.

As a first approach, we simplify our dataset to two classes by merging all degrees of flyers. This also eliminates normalization bias, since intensity values are no longer used—only the detection of the peptide is considered. Consequently, the training task becomes binary classification (in contrast to pFly, where binary labels are aggregated from categorical labels). These binary labels are sufficient to filter the search library, as we will show in the subsequent application.

On the other hand, we directly use the relative intensity as the label. To achieve this, we fine-tune the model through a regression task, predicting the relative intensity. For regression, we use the mean squared error of the relative intensity as the loss function. Classes can then be obtained a posteriori by applying a decision threshold. For these regression-based fine-tuning tasks, we limit ourselves to binary classes in order to maintain a common metric for comparison with the binary classification training.

Results are reported in Table 1. All models have been trained and evaluated on Zeno dataset splits. Regression-based fine-tuning achieved optimal performance when a higher proportion of non-flyers samples were included in the training set. Test datasets are binary balanced and identical across all setups. For regression task, the threshold has been chosen to optimize the accuracy on an independent validation set. Although overall accuracy differences between binary classification and regression tasks are minor, the types of errors differ significantly. Binary training task obtains significantly higher recall while regression shows a higher specificity. Depending on the intended use case and the consequences of errors, this balance can serve as an important criterion for model selection. For our purpose of filtering a search library, keeping undetectable peptides in the library is less harmful than removing detectable ones (we are directly losing probable identifications by removing them). The binary training task seems more suitable.

**Table 1:** Performance of alternative fine-tuning tasks on Zeno the test set.^a^

Task	Categorical	Binary	Regression
Astral			
Binary Acc	**0.685 ± 0.006**	0.679 ± 0.000	0.661 ± 0.012
Recall	0.905 ± 0.013	**0.980 ± 0.003**	0.857 ± 0.051
Specificity	**0.465 ± 0.005**	0.377 ± 0.003	0.464 ± 0.029
Zeno			
Binary Acc	**0.705 ± 0.004**	0.694 ± 0.003	0.693 ± 0.006
Recall	0.894 ± 0.009	**0.964 ± 0.006**	0.839 ± 0.019
Specificity	0.515 ± 0.005	0.421 ± 0.003	**0.545 ± 0.010**

a A model fine-tuned via categorical classification on the same dataset is included as a reference. “Categorical” and “Binary” refer to classification tasks, while “Reg $I_{\mathrm{rel}}$” refers to the regression task. For regression, the proportion of flyers was set to 75% during training; for classification tasks, it was 50%. Test datasets are always binary balanced (50% flyers, 50% non-flyers), which explains differences in binary accuracy compared to Figs 3 and 4, where categorical balanced test datasets were used.

### 4.5 Application to DIA library reduction

In a proteomic analysis pipeline using a spectral library, the size of the library strongly influences both computing time and the quality and number of identified peptides. Detectability can be leveraged to reduce library size by removing peptides that are unlikely to be detected. We illustrate this application in a bacterial identification pipeline, where the goal is to identify the species present in an unknown mixture of bacteria from a panel of 980 candidate species (typically for a medical diagnosis). The analysis is performed using DIA-NN 1.9.1 (Demichev *et al*. 2020). The test sample is a mixture of 12 species, with the exact composition detailed in the appendix. Experimental spectra were acquired with ZenoTOF. The large number of bacteria in the panel makes using a full proteome library impractical, as such a library would be too large for DIA-NN. Therefore, we first reduce the library to include only ribosomal proteins, elongation factors (tuf, tsf, fusA), and chaperonins (GroES, GroEL) from the human gut bacteria described by Almeida *et al*. (2021) and the human gut fungi described by Nash *et al*. (2017), along with the usual contaminants database (Frankenfield *et al*. 2022). This resulting library contains 783 304 precursors and is referred to as the “full library.”

**Figure 5:**
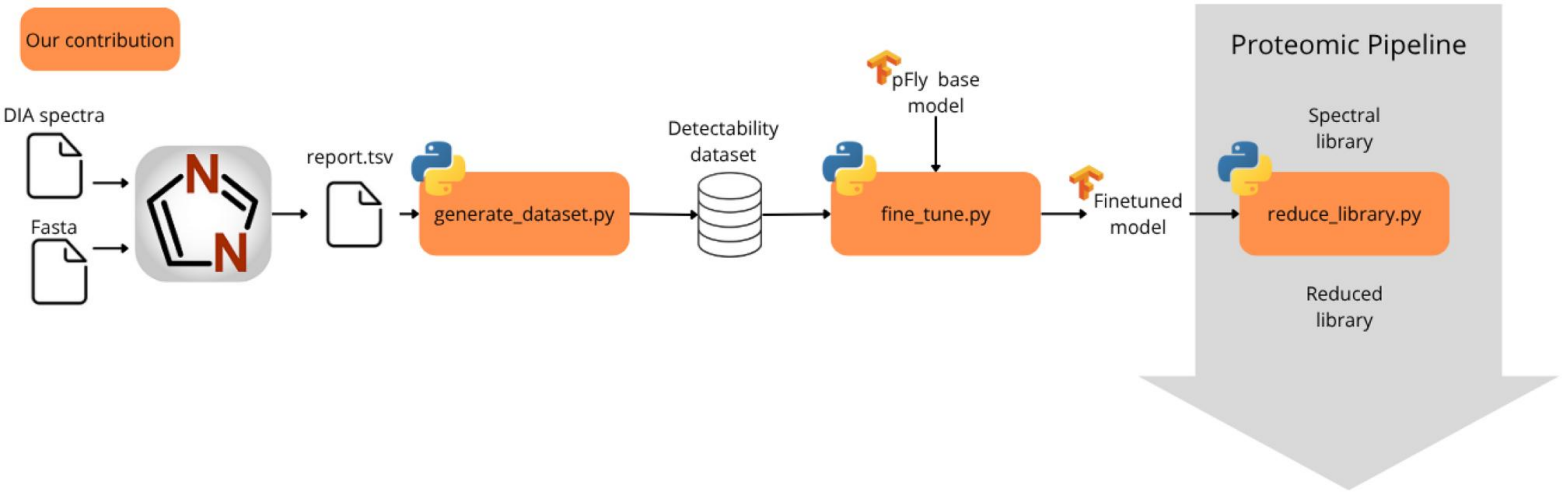
Schema of the complete library reduction pipeline. Python programs used to generate datasets, fine-tune detectability models, and reduce libraries are available at https://github.com/leoschn/Detectability.

The second step involves peptide detectability. We predict the detectability of peptides in the library using one of the detectability models. Peptides are then sorted by detectability, from lowest to highest, and nine reduced libraries are generated, containing the top 90%, 80%, …down to the top 10% most detectable peptides. Each of these reduced libraries is used independently to analyze the same raw experimental spectrum with DIA-NN. The complete pipeline is illustrated in Fig. 5. In the following experiment, we compared reduced libraries generated using pFly model with libraries generated using a model fine-tuned on our detectability dataset (binary task on Zeno dataset, see Table 1). As a baseline, we also include libraries reduced by randomly selecting peptides from the full library. For the fine-tuned and random models, we trained five independent models and report the mean number of peptide identifications (not applicable for baseline model since its weights are frozen).

**Figure 6:**
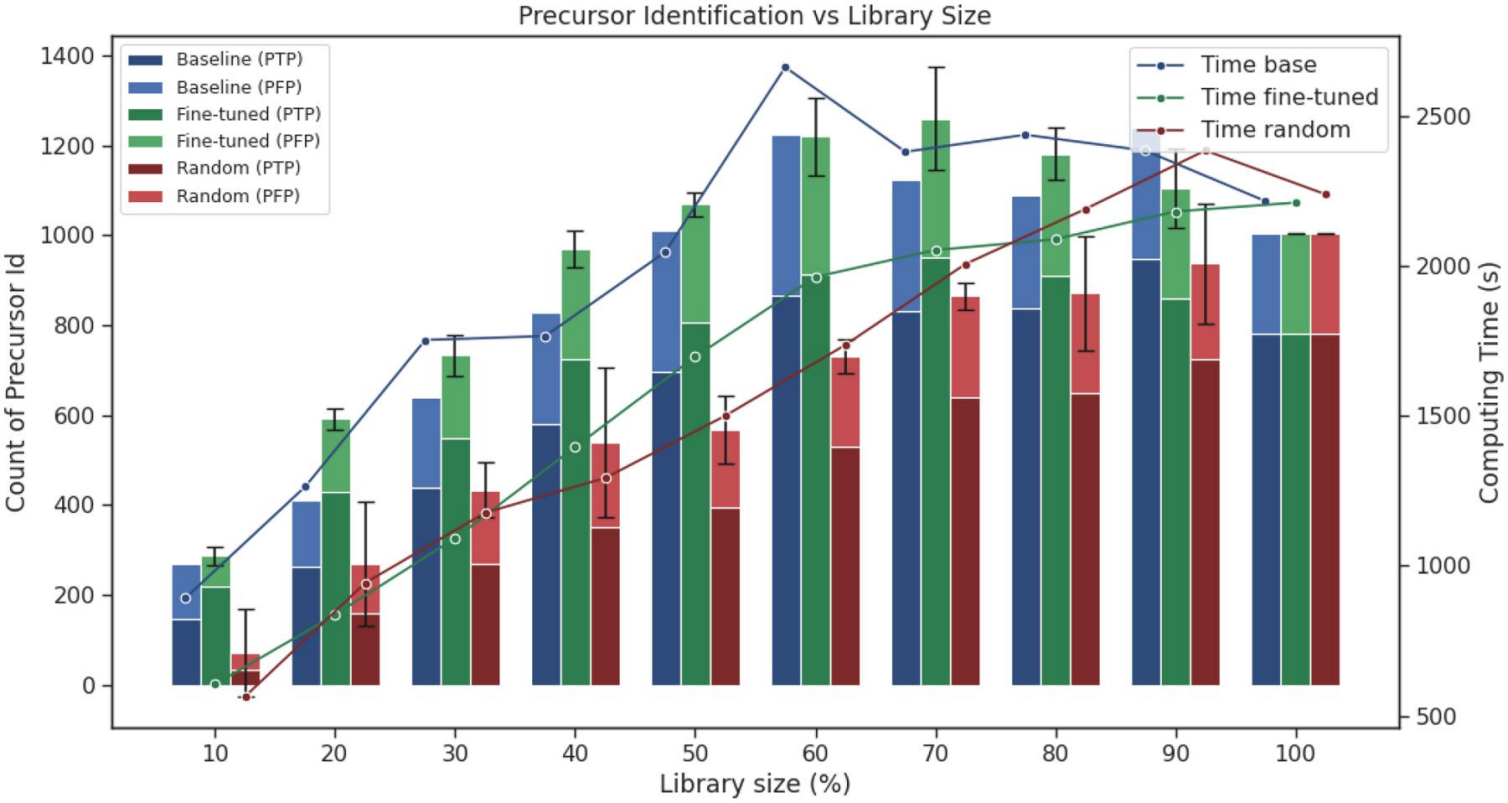
Library reduction results. Bars show the number of precursors identified for each reduced library: base model detectability in blue, fine-tuned detectability in green, and random reduction in red. 100% corresponds to the full library. Standard deviations for three replicates are shown for the fine-tuned and random libraries. Dark hue shows probable true positive (PTP) while light one relates to probable false positive (PFP) based on the presence of the sequence in one of the reference proteome of species present in the mix. The line indicates the total execution time of the peptide search by DIA-NN.

Results are shown in Fig. 6. Using the full library yields 1006 unique precursor identifications in 2216 s; this is identical across methods since no peptides are removed. For the random baseline, reducing the library gradually decreases the number of identifications, dropping to only 71 precursors on average for the smallest library. In contrast, libraries reduced based on detectability show more favorable results. Both detectability models yield more identifications for certain reduced libraries. The reduced libraries outperform the full library up to a 50% reduction. For example, removing the 30% least detectable peptides according to the base and fine-tuned models results in 1125 and 1259 identifications, respectively, corresponding to increases of 11% and 25%. Regarding computing time, smaller libraries generally lead to faster analyses. The fine-tuned model consistently achieves lower computing times than the base model. Note that these times do not include training, fine-tuning, or inference of the detectability model. Although the base model achieves a higher peak of identifications at 10% reduction, it is outperformed by the fine-tuned models for all the other reduction factors. We also investigated the quality of identifications. We considered identified precursors unrelated to any protein present in the reference proteome of the 12 species of the mix or to contaminants as probable false positive (reference proteomes downloaded on UniProt on 17_03_26). These identifications are displayed in light hues in the figure. It highlights that the probable false positive rate remains stable across library, new identifications are not due to more false positive.

**Figure 7:**
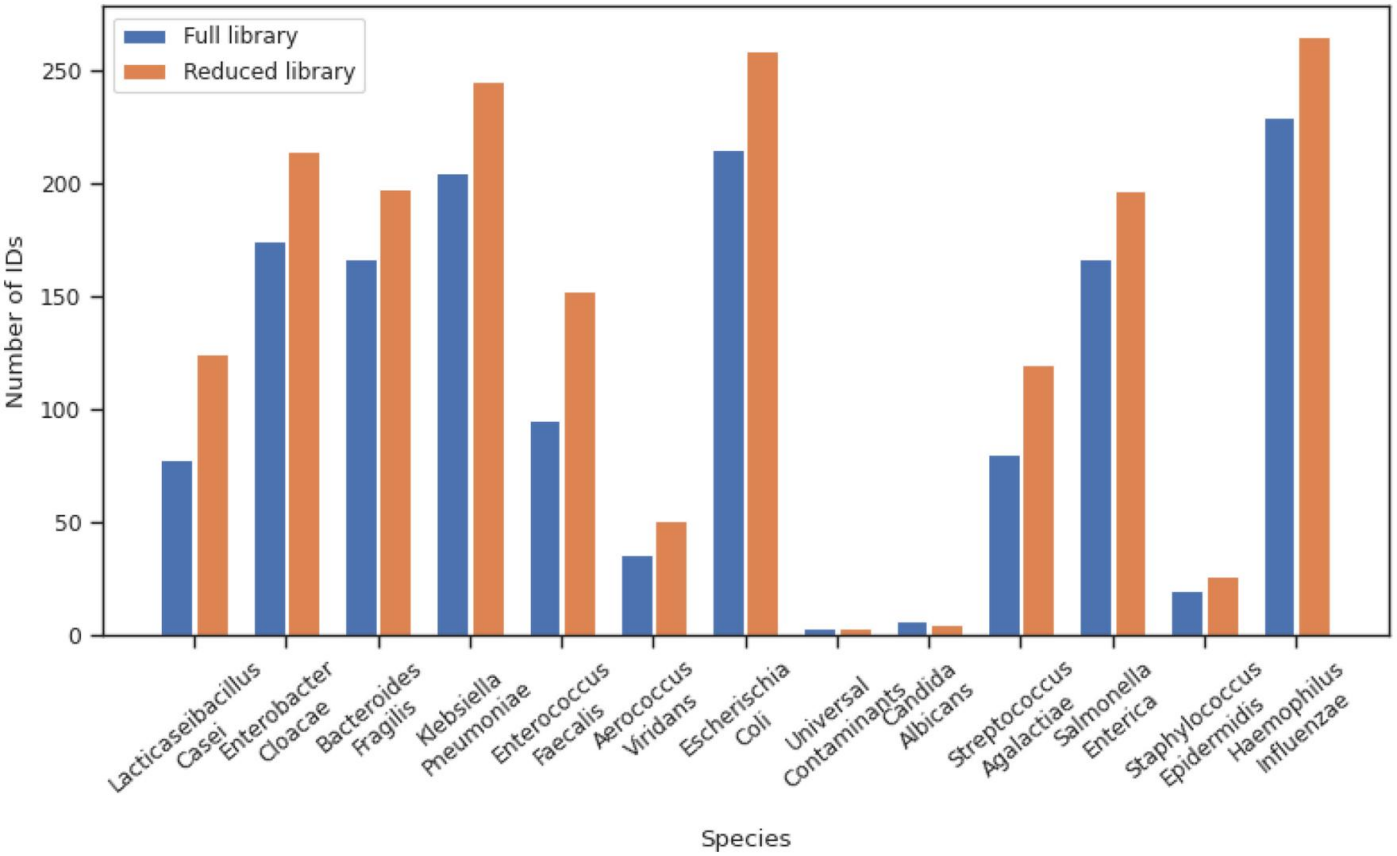
Number of identified peptides matching to each species present in the sample or contaminants with the full library and a library reduced by 30% with fine-tuned model.

We reported the species repartition among the probable true positive identifications in Fig. 7. In this analysis, the reduced library relates to one of the replicates with 30% reduction. If a peptide matches to several species proteomes at once it is counted in each one. The distribution of peptides matching each specie is uneven. Among the 12 species, 7 show >150 unique matches with the reduced lib while Candida Albicans shows almost no match. Interestingly we see that the repartition is similar with full and reduced library. Species in common between the sample used to extract the dataset used in fine tuning and this sample (for instance *Escherichia Coli* and *Bacteroides fragilis*, full list is reported in Appendix) does not report any additional increase in comparison to new species such as *Klebsiella pneumoniae*. The library reduction does not add biases toward some species.

**Figure 8:**
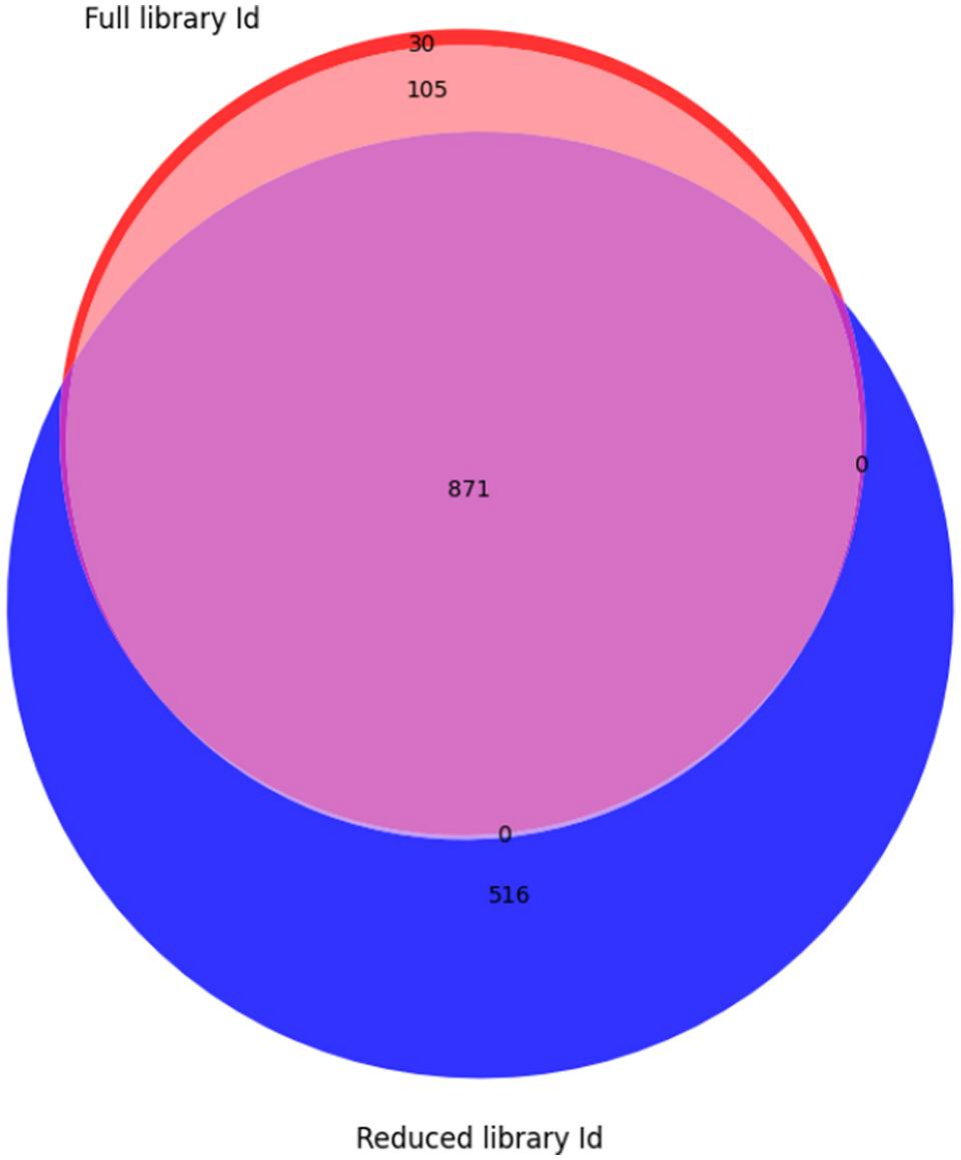
Venn diagram of precursors identified with the full library and a library reduced to 70 % based on our fine-tuned detectability model. Precursors in the pink intersection (105) were identified by the full library but not by the reduced library, even though they remain in the reduced search library. Precursors in the intersection (871) were identified by both the full and reduced libraries.

We also examined in greater detail the differences between identifications obtained with the full library and those obtained after removing 30% of the library based on our fine-tuned detectability model. In the Venn diagram (Fig. 8), 135 precursors are no longer identified after the reduction, while 516 new identifications compensate. Of the 135 missing precursors, 105 were removed from the library and are therefore not detected because they are no longer included. This highlights a limitation of the detectability model: some peptides that are actually detectable are still removed, indicating room for improvement. Interestingly, 105 precursors that remain in the reduced library are not detected, even though they were identified in the full library. Similar dynamics are observed across the other replicates. On average across all libraries reduced up to 50% by the fine-tuned model, these missing identifications show a probable false positive rate of 49% against 19% for the other identification with the full library. Their average q-value is also higher at 0.0029 against 0.0019. Both of these indicator suggest that a large part of these missing identifications were dubious ones in the first place.

## 5 Discussions

### 5.1 Normalization bias

The normalization of proteotypic peptide intensities within each protein clearly introduces a bias in our measurements. Since the intensity of a theoretically perfectly detectable peptide is unknown, we must rely on the most detectable peptide in each protein. The minimum peptide number per protein serves as an indirect way to mitigate this bias. Assuming that peptide detectabilities within a single protein are independent, the more peptides a protein has, the less likely it is that all are of low detectability. Therefore, increasing the minimum peptide-per-protein threshold in the labeling method can reduce this bias, although at the cost of decreasing dataset size.

**Figure 9:**
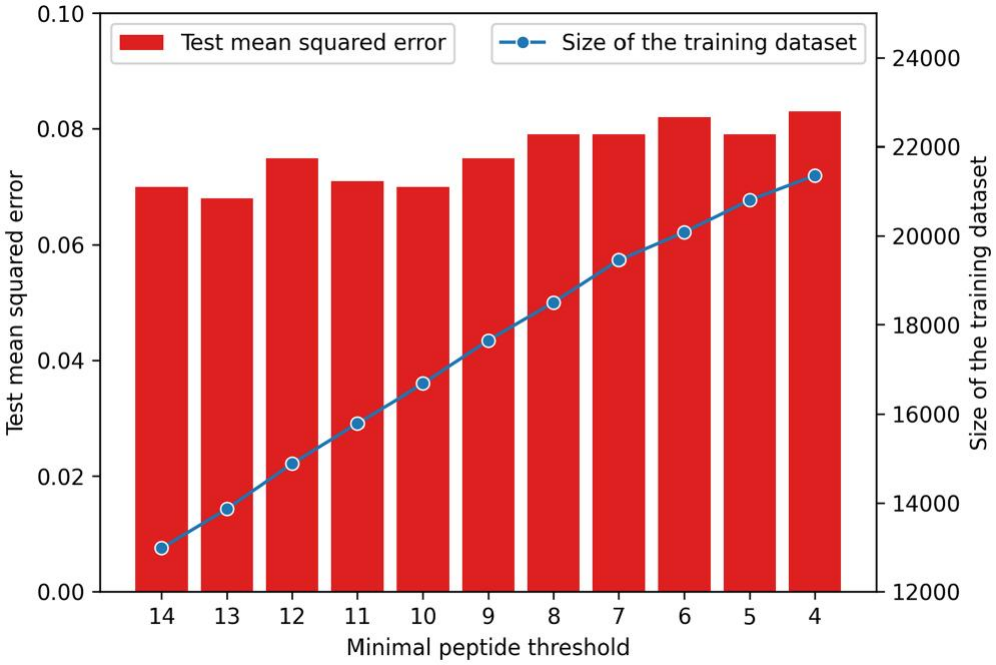
Mean squared error on the test set during detectability model fine-tuning, depending on the minimum peptides-per-protein threshold used to build the detectability dataset. The size of the corresponding training datasets is shown as a line.

We tested the impact of this threshold on model performance using the Astral dataset. Various minimum-peptide-per-protein thresholds were applied when extracting detectability, and regression training was used, as this protocol directly incorporates intensity values (normalization has no impact on binary models). Higher thresholds reduce normalization bias but also shrink the dataset. Results are shown in Fig. 9. Increasing the threshold clearly reduces dataset size while slightly reducing mean squared error. It is worth noticing that accuracy on binary prediction (not displayed) is stable across all minimal peptide threshold (0.80–0.81).

One hypothesis is that the larger dataset size compensates for the reduction in normalization bias, maintaining overall model performance.

### 5.2 Training dataset overlap with application library

In the application part, we demonstrate a realistic practical use case of the detectability filtering strategy. In real proteomics workflows, a detectability model would typically be trained on experimental datasets and subsequently applied to large spectral libraries that are likely to share at least some peptides with the training data. Such overlap is difficult to avoid entirely because many peptides are shared even across species and even across proteins. In our case, only 0.16% of the spectral library peptides were also present in the training set, which we consider representative of a realistic deployment scenario.

**Figure 10:**
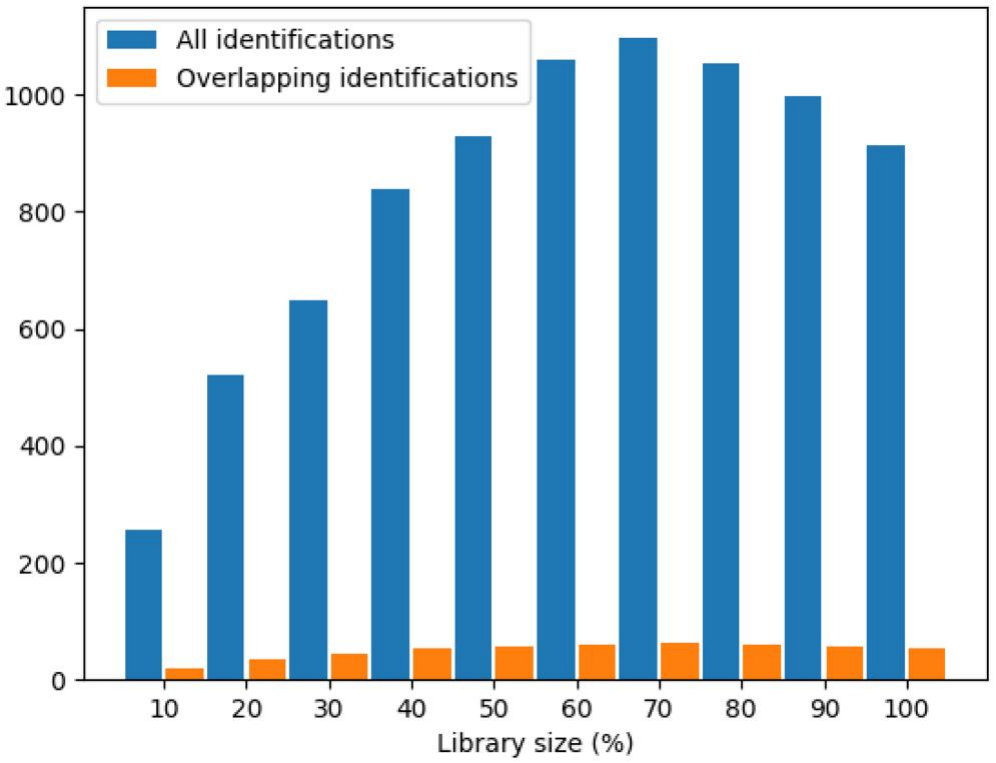
Number of identified peptides present in the fine-tuning dataset among all identified peptides for various library sizes. Average values over 5 replicates are displayed.

Nevertheless, we directly evaluated whether the observed performance improvement could primarily be explained by peptides overlapping with the training data. We found that peptides present in the training set represented a similar proportion of identifications obtained using the full library (5.8%) and the reduced libraries (5.7%–7.5% in average). Importantly, we remind that the full-library search does not involve detectability-based filtering. We display proportion of overlapping peptides among all identified peptides in Fig. 10. Moreover, for the library reduced by 30%—which yielded the highest number of additional identifications—in average only 11.4 of the 319 gained identifications (3.6%) corresponded to peptides present in the training set. Therefore, overlapping peptides are not overrepresented among the gained identifications relative to the baseline search.

This analysis indicates that the mere presence of overlapping peptides is not sufficient to explain the improvement and confirm the utility of detectability fine-tuning for library reduction.

## 6 Conclusion

In this article, we propose a new method to extract peptide detectability scores from a single DIA experiment, based on the relative intensities of proteotypic peptides. Using these scores, we fine-tuned the state-of-the-art detectability prediction model pFly for a specific acquisition platform. We also explored and evaluated several fine-tuning tasks, ranging from regression to binary classification. Our results show that fine-tuning with such datasets improves model transferability without requiring large amounts of raw data.

As an application, we demonstrate that filtering a search library based on detectability increases the number of peptide identifications and reduces computing time. Both the base and fine-tuned detectability models exhibit complementary strengths: the base model performs best for slight library reductions, while the fine-tuned model excels for more drastic reductions and faster analyses. Depending on the use case, both models can be valuable. In our experiments, removing up to 50% of the library yielded more identifications than the full library.

By providing a data-efficient method to transfer peptide detectability to a specific proteomics platform, we hope that detectability will be increasingly integrated into proteomics pipelines. This approach can be adapted to other deep learning-based detectability models. As shown in our application, detectability has the potential to optimize proteomics workflows.

Future work will focus on further improving performance and exploring optimal architectures for enhanced transferability.

## Supplementary Material

vbag180_Supplementary_Data
