# Supplementary material for: Generation of peptide detectability datasets from single DIA experiment for prediction model fine-tuning
Source: Bioinform Adv. 2026 Jun 25;6(1):vbag180. doi: 10.1093/bioadv/vbag180 (PMC13407425; doi:10.1093/bioadv/vbag180)
Supplement: vbag180_Supplementary_Data [file vbag180_supplementary_data.zip › appendix_detectabilité_template_bioinformatics__cleaned_v3.pdf]

Detailed LC-MS methods

The mass spectrometry proteomics data have been deposited to the ProteomeXchange Consortium via the PRIDE Perez-Riverol et al. 2025 partner repository with the dataset identifier `PXD076276`.

Sample preparation

Zeno dataset

The sample used to generate specific flyer/non-flyer labels for the Zeno dataset was the ZymoBIOMICS®Gut Microbiome Standard from Zymo Research (catalog number: D6331). This sample is composed of 21 different strains from 17 different micro-organisms at different concentrations (*Clostridium perfringens*, *Enterococcus faecalis*, *Salmonella enterica*, *Candida albicans*, *Saccharomyces cerevisiae*, *Methanobrevibacter smithii*, *Clostridioides difficile*, *Akkermansia muciniphila*, *Escherichia coli*, *Prevotella corporis*, *Fusobacterium nucleatum*, *Bacteroides fragilis*, *Bifidobacterium adolescentis*, *Lactobacillus fermentum*, *Roseburia hominis*, *Faecalibacterium prausnitzii*, *Veillonella rogosae*). The sample was thawed and then vortexed the day of the analysis.

Astral dataset

The sample used to generate specific flyer/non-flyer labels for the Astral dataset was a home-made micro-organism mixture composed of 12 different micro-organisms (*Aerococcus viridans*, *Bacteroides fragilis*, *Candida albicans*, *Enterobacter cloacae*, *Enterococcus faecalis*, *Escherischia Coli*, *Haemophilus influenzae*, *Klebsiella pneumoniae*, *Lacticaseibacillus casei*, *Salmonella enterica*, *Staphylococcus epidermidis*, *Streptococcus agalactiae*) at different concentrations. The strains were first grown on appropriate agar for 48h before adjusting concentration to 0.5 to 3 McFarland in 0.45% NaCl. The detailed preparation can be found in the Supplementary Table 1. The same sample is used in the application after being analysed with ZenoTOF.

For both samples

The tube was then centrifuged for 5 min at 16100 g, the supernatant discarded, and the pellet resuspended in 1mL LC–MS/MS grade water before being transferred into a new 1.5 mL tube. Then, the tube was centrifuged again (5 min, 16100 g) and the supernatant discarded. Next, 200 µL of LC–MS/MS-grade water was added to the pellet as well as approximately 70 mg of glass beads (acid washed, 150-212 µm, SigmaAldrich, St Louis, MO, USA). Bacterial lysis and protein digestion were performed simultaneously by adding 5 µL of 1 mg/mL trypsin (Roche Diagnostics, Mannheim, Germany) prepared in a 150 mM NH4HCO3 solution. The mixture was then placed in a thermostated (50 °C) ultrasonic bath (Bioruptor Plus, Diagenode, Liège, Belgium); ultrasounds were applied at low power for 30 cycles (30 s on – 30 s off). Trypsin digestion was then stopped by adding 5 µL of pure formic acid. Digests were finally centrifuged for 5 min at 9600 g and 100 µL of supernatant was transferred into the final screw cap vial.

K562 replicates

The purchased protein digest standard was K562 MS Compatible Human Protein Extract, Digest, 100µg (Promega). Working stocks were prepared adding 1mL of 0.1% formic acid in 98% LC-MS grade water and 2% LC-MS grade acetonitrile, aliquoted, and stored at –20○C until further use.

ZenoTOF

In the analysis using the ZenoTOF 7600+ (SCIEX), peptides were separated at a flow rate of 2 $\mu L\,min^{-1}$ on an Evosep Endurance EV1106 column (15 cm × 150 µm, 1.9 µm) using the ACQUITY M-Class UPLC system (Waters). Mobile phase A consisted of 100% water with 0.1% formic acid (FA), and mobile phase B was 100% acetonitrile with 0.1% FA.

The chromatographic method began at 2% B, followed by a short gradient from 2 to 10% B over 1 minute, then a gradient from 10 to 40% B over 44.5 minutes. The column was washed with 98% B for 5 minutes and re-equilibrated to 2% B for 5 minutes, resulting in a total analysis time of 55 minutes. The column temperature was maintained at 40°C, and the sampler at 5°C.

Data were acquired in positive mode with a source voltage of 4500 V. Ion source gas 1 pressure was set to 15 psi, ion source gas 2 to 60 psi, curtain gas to 35 psi, and CAD gas to 7 psi, with a source temperature of 150°C. MS1 scans ranged from 325 to 1500 m/z with an accumulation time of 50 ms. The declustering potential was 80 V, and the collision energy was set to 10 V.

Peptides were fragmented using CID, with MS2 scans ranging from 200 to 1800 m/z and an accumulation time of 10 ms, using 65 variable m/z windows for precursor isolation. The corresponding mass table is provided in Supplementary Table. The total scan time was 1.098 s, and signal intensities were derived by summing signals over 8-time bins.

Orbitrap Astral

For the analysis on the Orbitrap Astral (Thermo Fisher Scientific), dissolved peptides were loaded onto a PepMap™ Neo 5 µm C18 trap column (300 µm × 5 mm) and separated on an Aurora Ultimate™ 25×75 µm C18 UHPLC column (75 µm × 25 cm, 1.6 µm, 120 Å; IonOpticks) using the UHPLC Vanquish™ Neo system (Thermo Fisher Scientific). Mobile phase A consisted of 100% water with 0.1% formic acid (FA), and mobile phase B contained 80% acetonitrile, 20% water with 0.1% FA.

The gradient was initiated at 0% solvent B. Solvent B was ramped to 4% at 0.14 min and 8% at 0.40 min with a flow rate of 500 nL/min. At 0.5 min, the flow rate was reduced to 400 nL/min. Solvent B was then gradually increased to 20% at 16.5 min, 30% at 22.5 min, and 45% at 26 min. At 27.6 min, the flow rate was returned to 500 nL/min, and solvent B was raised to 99% for 30 min.

Eluting peptides were subsequently analysed on the Orbitrap Astral. MS1 spectra were acquired over m/z 380–980 at a resolution of 240,000, with an automatic gain control (AGC) target of 500% and a maximum injection time (MIT) of 5 ms. MS2 spectra were collected in the m/z range 150–2,000 using the Astral analyzer, with an AGC of 500%, MIT of 3 ms, and a normalized collision energy of 25%. MS2 precursor isolation width was set to 2 Th, with window placement optimization enabled.

K562 replicates

The analysis was run on the ZenoTOF 7600+ with an OptiFlow source with the SteadySpray Low Micro Electrode (SCIEX) connected to an ACQUITY M-Class UPLC (Waters). The peptides were separated on a 150 ×0.3 mm Kinetex 2.6 µm XB-C18 column (Phenomenex) that was heated to 30○C while running at 5 $\mu L\,min^{-1}$. The chromatographic method began at 3% B for 1 min, followed by a gradient from 3 to 30% B

over 20 minutes. The column was washed with 80% B for 2 minutes and re-equilibrated to 3% B for 5 minutes, resulting in a total analysis time of 30 minutes. Data were acquired in positive mode with a source voltage of 5000 V. Ion source gas 1 pressure was set to 20 psi, ion source gas 2 to 60 psi, curtain gas to 35 psi, and CAD gas to 7 psi, with a source temperature of 200°C. MS1 scans ranged from 400 to 1500 m/z with an accumulation time of 10 ms. The declustering potential was 80 V, and the collision energy was set to 10 V. Peptides were fragmented using CID, with MS2 scans ranging from 140 to 1750 m/z and an accumulation time of 13 ms, using 65 variable m/z windows for precursor isolation. The corresponding mass table is provided in Supplementary Table. The total scan time was 1.283 s, and signal intensities were derived by summing signals over 8-time bins.

Escherichia coli sample

Bacterial isolation and sample preparation

1 mL of positive blood culture was withdrawn from the bottle and transferred to a 1.5 mL Protein LoBind tube (Eppendorf). 200 µL of a 6% sodium dodecyl sulfate (SDS) solution was added, and the tube was vortexed for 10 seconds, followed by centrifugation (2 minutes at 16,100 g). The supernatant was then discarded, and the pellet was resuspended in 1 mL of LC-MS/MS-grade water (Thermo Fisher Scientific). The tube was centrifuged again (1 minute at 16,100 g), then the supernatant was discarded and the pellet resuspended once more in 1 mL of LC-MS/MS-grade water before being transferred to a new 1.5 mL Protein LoBind tube. The latter was centrifuged again (1 minute, 16,100 g), and the supernatant was discarded. To enhance bacterial lysis, one spoonful of acid-washed glass beads (150–212 µm, Sigma-Aldrich) was added to each sample. For protein digestion, a 1 mg/mL trypsin solution was prepared in 150 mg ammonium bicarbonate buffer using a 68 mg/mL Roche liquid trypsin solution. Subsequently, 50 µL of this trypsin solution was added to each tube containing the glass beads and the bacterial culture.

The tubes were then placed in a Bioruptor sonicator (Diagenode) set to 50°C, where they underwent 10 cycles of one minute each (30 seconds of ultrasound ON, 30 seconds of ultrasound OFF). Digestion was stopped by acidifying the samples with 5 µL of formic acid. Finally, the digestates were centrifuged at 9,600 g for 5 min, and 100 µL of the supernatant was transferred to a 1.5 mL amber screw-cap vial. All steps involved in preparing blood cultures, isolating bacteria, and preparing tryptic hydrolysates were performed by Chloé Desbiolles, Roxane Prat, and Francis Deforet.

LC-MS/MS Analysis

LC-MS/MS analyses were performed in Zeno SWATH DIA acquisition mode using the optimized 100-variable-window method on a M-Class UPLC liquid chromatography system (Waters) coupled to a ZenoTOF 7600 mass spectrometer (Sciex) equipped with an OptiFlow TurboV source. Peptides were separated on an HSS-T3 column (300 µm × 150 mm; particle diameter 1.8 µm) (Waters) heated to 40°C. Separation was performed using water containing 0.1% (v/v) FA as solvent A and acetonitrile containing 0.1% (v/v) FA as solvent B, following this elution profile at 6 $\mu L\,min^{-1}$: 2%–10% B in 0.1 min, 10%–50% B in 11.5 min, 50%–98% B in 0.1 min. The final step consisted of a wash plate at 98% B for 5.4 min. The gradient was then returned to the initial conditions and maintained for 7.5 min. The bacterial hydrolysate was diluted 10-fold, and 1 µL of this solution was injected for LC-MS/MS analysis. LC-MS Analysis was performed by Iulia Macavei.

Application

The 12 species sample (same sample as the Astral dataset) was analysed with the Zeno platform for the application. Data acquisition protocol is identical to the one described in the ZenoTOF part.

Data processing

Raw data were analysed using DIA-NN v1.9.1 with a search library composed of full proteomes and common contaminants, using a 350–1250 m/z search window. Methionine oxidation was set as the only variable modification, and one missed cleavage was allowed.

The full setting for the dataset extraction is the following : (–qvalue 0.01 –matrices –gen-spec-lib –predictor –fasta-search –min-fr-mz 200 –max-fr-mz 1800 –min-pep-len 5 –max-pep-len 30 –min-pr-mz 350 –max-pr-mz 1250 –min-pr-charge 1 –max-pr-charge 4 –cut K*,R* –missed-cleavages 1 –var-mods 1 –var-mod UniMod:35,15.994915,M –reanalyse –relaxed-prot-inf –rt-profiling). For the application (final search with reduced database) the same settings have been used but –var-mods 0.

For the Zeno dataset, the 17 reference proteomes corresponding to the 17 species were downloaded from UniProt on 05_03_2025 and combined with the standard contaminants database (downloaded on 27_07_2024 from Frankenfield et al. 2022, `https://github.com/HaoGroup-ProtContLib`). For the Astral dataset, the 12 reference proteomes from the 12 species were downloaded from UniProt on 22_01_2025 and combined with the same contaminants database.

Peptides were filtered according to the following criteria. Low-quality identifications were removed (q-value $\geq$ 0.01). To match the equal concentration assumption, peptides with one or more missed cleavages and non-proteotypic peptides were excluded. Proteotypicity was computed based on the FASTA used as the search library during the DIA-NN analysis. Since no measures were taken to prevent disulfide bridge formation during sample preparation, peptides containing cysteine were also removed. Finally, to limit normalization bias, only peptides from proteins with a $\geq$ 20% sequence coverage and at least 4 identified peptides (unless stated otherwise),and with a length of at least 5 were retained.

For flyers, identified peptides were grouped by protein, and their intensities were normalized within each protein. All normalized intensities values were then sorted: peptides in the top tertile were classified as strong flyers, those in the middle tertile as intermediate flyers, and those in the bottom tertile as weak flyers. The quantification method used to get this intensity in the main paper was based on MS2 intensity (*Fragment.Quant.Raw* DIA-NN output column. We compute the intensity as the sum of all fragment intensity. When multiple precursors share the same sequence but with different charges, we only retain the top intensity one. Other quantification methods have been tested namely :

- MaxLFQ quantification for peptides (we use regular MaxLFQ method but consider each peptide as independent proteins)
- MS1 quantification based on *Precursor.Quantity* DIA-NN output column.

Experiment	sample	instrument	peptide th	total size	train flyer prop	val/test flyer prop
Fine-tuning Astral	12 mix	Astral	4	26706	75%	75%
Fine-tuning Zeno	17 mix	Zeno	4	6862	75%	75%
Alternative tasks	17 mix	Zeno	4	7548	75%	50%
Application model	17 mix	Zeno	4	7548	75%	50%
Normalisation bias	12 mix	Astral	variable	variable	75%	75%

Table 1. Summary of all datasets configurations.

- for MS1 and MS2 quantification we also try to aggregate precursors with same sequence but different charges by summing their intensity.

A stability analysis reported in this appendix compares all 5 methods B.

We acknowledge that this normalization introduces bias, as the most intense peptide in each protein is assumed to be a perfect flyer and used as a reference. To mitigate this bias, a minimum number of peptides per protein threshold was applied.

For non-flyers, unidentified peptides from identified proteins were selected. This typically results in more non-flyer peptides than the total number of flyers. To maintain a balanced dataset, only a fraction of non-flyers equal in number to the flyers was retained.

For the application final search (not application model fine-tuning), we used the same 12-species sample as in the Astral training dataset, but analysed on the Zeno platform. The FASTA file differed, containing ribosomal proteins, elongation factors (tuf, tsf, fusA), and chaperonins (GroES, GroEL) from the human gut bacteria described by Almeida et al. 2021 and the human gut fungi described by Nash et al. 2017, assembled with the standard contaminants database.

In Tab. 1 we summarize all configuration of datasets used for training and evaluation models in the paper. Train, validation and test splits proportion are 0.8, 0.1 and 0.1. These proportions might vary slightly due to protein-level random split used.

Detectability stability

Detectability as we measure it is not an intrinsic property of each peptide. As stated before it depends on multiple factors, ranging from digestibility to instrumental setup which motivates fine-tuning of the prediction model for each setup. To validate the quality of our measurement method we computed detectability from 4 replicates of DIA analysis of the same samples and compared it. We display the results as pairwise scatter plots with their Pearson's correlation value for each quantification method in Fig. 1, 2, 3, 4 and 5. Each pairwise comparison shows a relatively high correlation for peptides detected in both experiments. The Person correlation value ranges from 0.76 to 0.82 and is the highest with MS2 intensity based quantification method.

We also show how the detectability intensity translates to label assignments in Fig. 6, 7, 8, 9 and 10.

This study shows that the most stable quantification method across replicates is maximum of MS2 intensity (fragments quantity based). It shows the highest mean pairwise correlation and the the highest mean number of peptides with the same labels assigned. This method was chosen for the paper. Even if the detectability from a single DIA experiment is sufficient for fine-tuning as our application showed, its variability could be reduced by using more experiments (replicates analysis

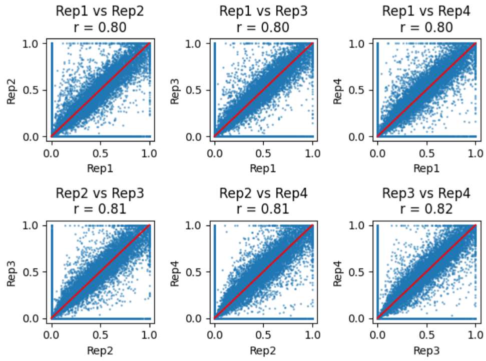

Fig. 1. Pairwise correlation between detectability measurement based on max fragment intensity from 4 K562 DIA replicates

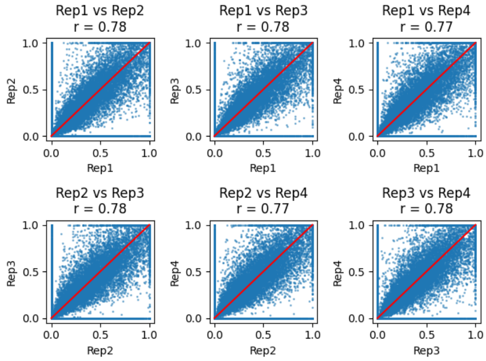

Fig. 2. Pairwise correlation between detectability measurement based on max precursor intensity from 4 K562 DIA replicates

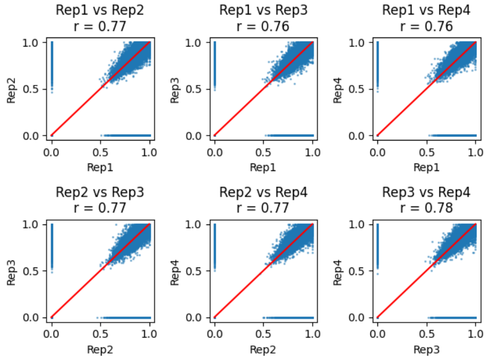

Fig. 3. Pairwise correlation between detectability measurement based on MaxLFQ intensity from 4 K562 DIA replicates

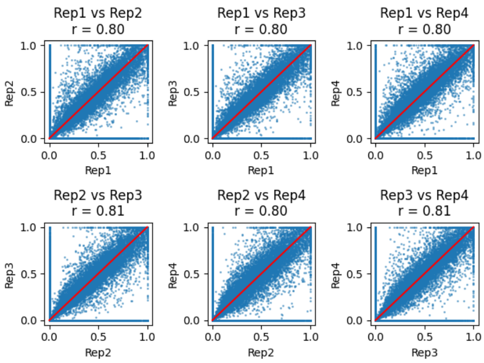

Fig. 4. Pairwise correlation between detectability measurement based on summed fragment intensity from 4 K562 DIA replicates

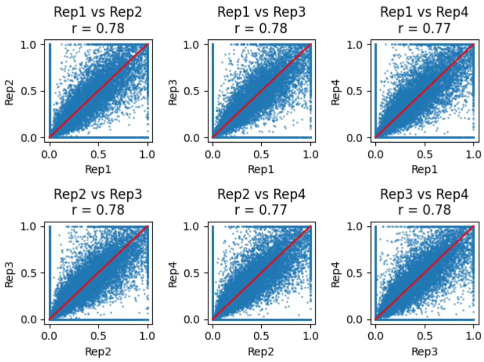

Fig. 5. Pairwise correlation between detectability measurement based on summed precursor intensity from 4 K562 DIA analysis replicates

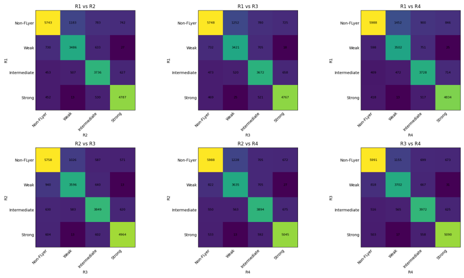

Fig. 6. Pairwise confusion matrix between assigned labels based on summed precursor intensity from 4 K562 DIA analysis replicates

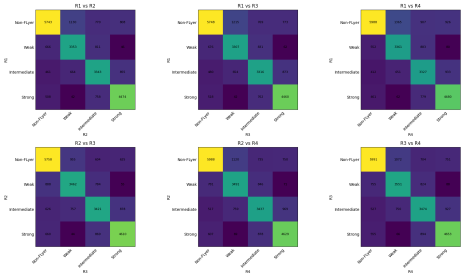

Fig. 7. Pairwise confusion matrix between assigned labels based on summed precursor intensity from 4 K562 DIA analysis replicates

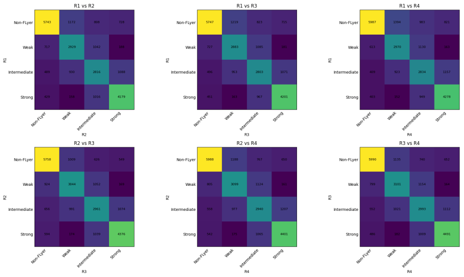

Fig. 8. Pairwise confusion matrix between assigned labels based on summed precursor intensity from 4 K562 DIA analysis replicates

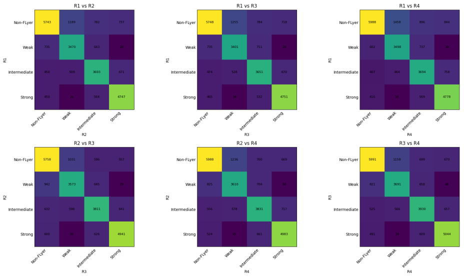

Fig. 9. Pairwise confusion matrix between assigned labels based on summed precursor intensity from 4 K562 DIA analysis replicates

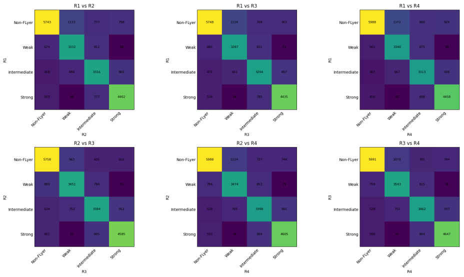

Fig. 10. Pairwise confusion matrix between assigned labels based on summed precursor intensity from 4 K562 DIA analysis replicates

of a single sample). When possible this strategy should be beneficial.

Model training parameters

Table 2 summarizes the parameters used for the different training experiments. All experiments employed an 80%–20% train-test split and were conducted without early stopping. For fine-tuning the base model was taken from pFly publicly available code (original_detectability_fine_tuned_model_FINAL).

For the alternative tasks (binary classification and regression), the output size of the last fully connected layer was adjusted from four to two and one, respectively. In these cases, the weights of this layer were initialized to zero.

Learning rate optimization

We defined learning rate by grid search. The grid search was performed on multiclass task on Zeno dataset. Results are displayed on Fig. 11. From scratch training showed

Table 2. Summary of parameters used for each model training.

Task	Epochs	Batch size	Loss function	Learning rate	Optimizer
Fine-tuning and transferability					
Multiclass	50	16	Cross Entropy	1×10^{-7}	Adagrad
Multiclass (from scratch)	50	16	Cross Entropy	5×10^{-2}	Adagrad
Alternative fine-tuning tasks					
Multiclass	50	16	Cross Entropy	1×10^{-7}	Adagrad
Binary	50	16	Cross Entropy	5×10^{-2}	Adagrad
Regression	50	16	Mean Squared Error	5×10^{-2}	Adagrad
Application					
Binary	50	16	Cross Entropy	5×10^{-2}	Adagrad

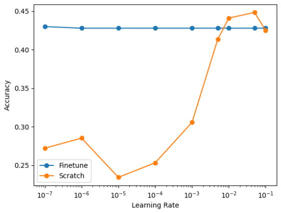

Fig. 11. Multiclass accuracy on Zeno dataset depending on the learning rate for training from scratch and fine-tuning after 50 epochs.

more sensitivity to learning rate value in comparison to fine-tuning. while fine-tuned model accuracy is stable, reaching top performances across all LR values, on the other hand model trained from scratch accuracy is close to random for low LR (10^{-7} to 10^{-3} and only reaches top performance for 5.10^{-3} to 5.10^{-2} LR.

Training curves

To avoid unnecessary redundancy and excessive supplementary figures, we report representative curves rather than all individual models, as a total of 140 models were trained during the study. The curves displayed in Fig. 12 are related to the model fine-tuned and Astral dataset with detailed labels (4 classes). While exact loss value slightly varies across datasets and replicates, dynamics remains similar.

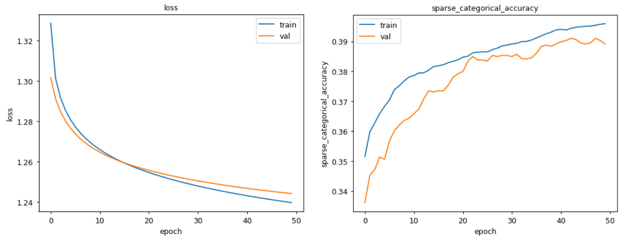

Fig. 12. Training and validation losses curves (left) and accuracy (right) of model fine-tuning on Astral dataset with multiclass labels (cross entropy loss function).

Detailed Fine-tuning results

We display here detailed results of fine-tuning experiment aggregated in Fig. 3-4 of the main paper.

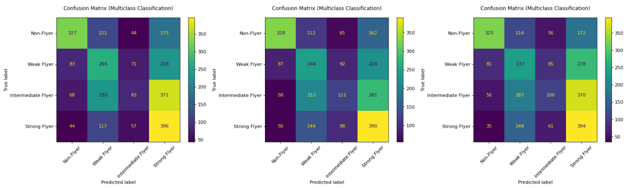

Fig. 13. Confusion matrix of model fine-tuned on Astral dataset and evaluated on Astral dataset (3 replicates).

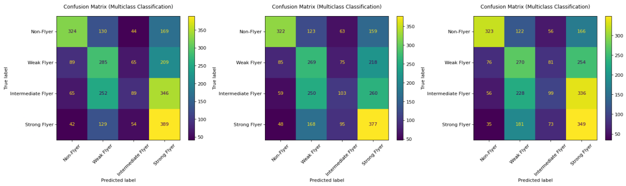

Fig. 14. Confusion matrix of model fine-tuned on combined dataset and evaluated on Astral dataset (3 replicates).

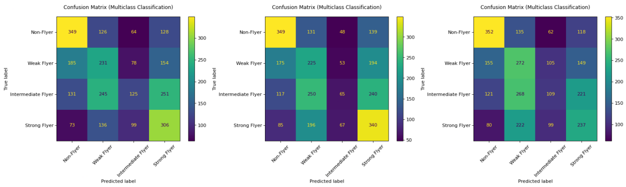

Fig. 15. Confusion matrix of model fine-tuned on Zeno dataset and evaluated on Astral dataset (3 replicates).

Application of Library Reduction to Additional Spectral Libraries

To assess the robustness of the library reduction strategy presented in the main manuscript, we replicated the analysis on two additional spectral libraries. Both libraries were

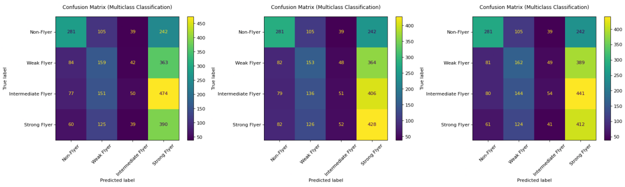

Fig. 16. Confusion matrix of pFly model evaluated on Astral dataset (3 replicates).

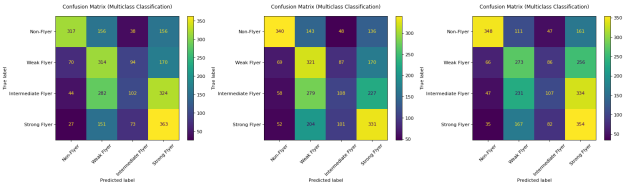

Fig. 17. Confusion matrix of model trained from scratch on Astral dataset and evaluated on Astral dataset (3 replicates).

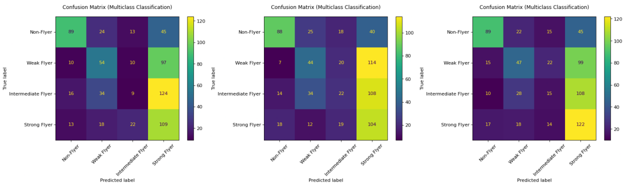

Fig. 18. Confusion matrix of model fine-tuned on Astral dataset and evaluated on Zeno dataset (3 replicates).

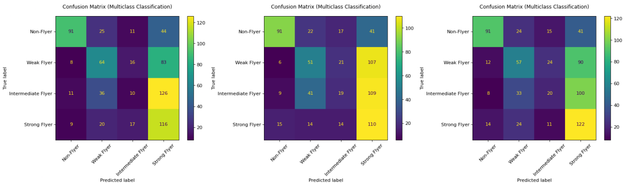

Fig. 19. Confusion matrix of model fine-tuned on combined dataset and evaluated on Zeno dataset (3 replicates).

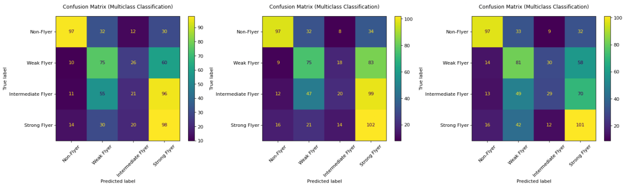

Fig. 20. Confusion matrix of model fine-tuned on Zeno dataset and evaluated on Zeno dataset (3 replicates).

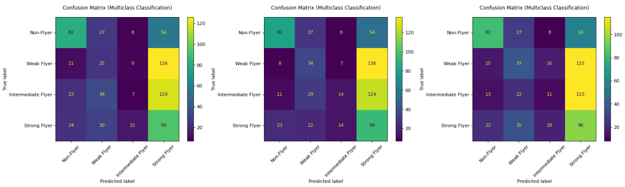

Fig. 21. Confusion matrix of pfly model evaluated on Zeno dataset (3 replicates).

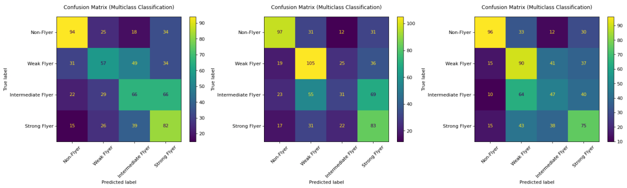

Fig. 22. Confusion matrix of model trained from scratch on Astral dataset and evaluated on Zeno dataset (3 replicates).

generated using DIA-NN with the same parameters as those used for the application dataset. The human library was constructed from the UniProt proteome UP000005640, and the *Escherichia coli* library from UniProt proteome UP000000625 (downloaded on 2026-06-10). The same fine-tuned models used in the main application was employed to compute peptide detectability scores, without any additional training or parameter modifications. The human library has been used to analyse a K562 sample (first replicate) while the the *E. coli* to analyze a sample containing *E. coli* only (preparations described Section A.2).

Figure 23 shows the number of precursor identifications obtained using libraries reduced according to peptide detectability and libraries reduced at random. Detectability-based reduction consistently yields a higher number of identifications than random reduction across all library sizes. It is also noteworthy that, unlike the human dataset and the application presented in the main manuscript, the reduced Escherichia coli libraries never outperform the full library. This observation may be explained by the relatively small size of the *E. coli* library compared with the other two, suggesting that additional library reduction provides limited benefit in case of small libraries.

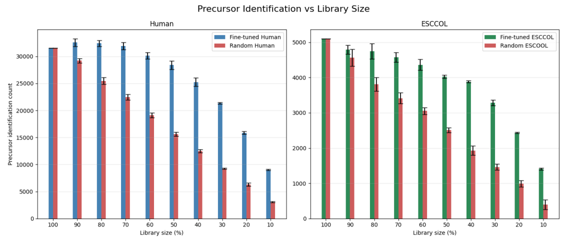

Fig. 23. Library reduction results. Bars show the number of precursors identified for each reduced library with DIA-NN: fine-tuned detectability in green (*E. coli*) and blue (human), and random reductions in red. 100% corresponds to the full library. Standard deviations for five replicates are shown for the fine-tuned and random libraries.

References

Perez-Riverol, Y C Bandla, DJ Kundu, S Kamatchinathan, J Bai, S Hewapathirana, NS John, A Prakash, M Walzer, S Wang, and JA Vizcaíno (2025) The PRIDE database at 20 years: 2025 update. *Nucleic Acids Research* 53 (D1) D543–D553.

Frankenfield, AM J Ni, M Ahmed, and L Hao (2022) Protein Contaminants Matter: Building Universal Protein Contaminant Libraries for DDA and DIA Proteomics. *Journal of Proteome Research* 21, 2104–2113.

Almeida, A S Nayfach, M Boland, F Strozzi, M Beracochea, ZJ Shi, KS Pollard, E Sakharova, DH Parks, P Hugenholtz, N Segata, NC Kyrpides, and RD Finn (2021) A unified catalog of 204,938 reference genomes from the human gut microbiome. *Nature Biotechnology* 39, 105–114.

Nash, AK TA Auchtung, MC Wong, DP Smith, JR Gesell, MC Ross, CJ Stewart, GA Metcalf, DM Muzny, RA Gibbs, NJ Ajami, and JF Petrosino (2017) The gut mycobiome of the Human Microbiome Project healthy cohort. *Microbiome* 5, 153.
